# The level of habitat patchiness influences movement strategy of moose in Eastern Poland

**DOI:** 10.1371/journal.pone.0230521

**Published:** 2020-03-19

**Authors:** Tomasz Borowik, Mirosław Ratkiewicz, Weronika Maślanko, Norbert Duda, Rafał Kowalczyk

**Affiliations:** 1 Mammal Research Institute Polish Academy of Sciences, Białowieża, Poland; 2 University of Białystok, Institute of Biology, Białystok, Poland; 3 University of Life Sciences in Lublin, Department of Animal Ethology and Wildlife Management, Lublin, Poland; 4 Zespół Szkół Ogólnokształcących Nr 2 w Białymstoku, Białystok, Poland; Universita degli Studi di Sassari, ITALY

## Abstract

Spatio-temporal variation in resource availability leads to a variety of animal movement strategies. In the case of ungulates, temporally unpredictable landscapes are associated with nomadism, while high predictability in the resource distribution favours migratory or sedentary behaviours depending on the spatial and temporal scale of landscape dynamics. As most of the surveys on moose (*Alces alces*) movement behaviours in Europe have been conducted on Scandinavian populations, little is known about the movement strategies of moose at the southern edge of the species’ range. We expected that decreasing habitat patchiness in central Europe would be associated with the prevalence of migratory behaviours. To verify this hypothesis, we analysed 32 moose fitted with GPS collars from two study areas in eastern Poland which differed in a level of habitat patchiness. We classified moose movements using the net squared displacement method. As presumed, lower patchiness in the Biebrza study site was associated with the predominance of individuals migrating short-distance, while in more patchy landscape of Polesie, resident moose dominated. At the individual level, the propensity of moose to migrate decreased with increasing abundance of forest habitats in their summer ranges. In addition, the parameters (migration distance, timing and duration) for migratory individuals varied substantially between individuals and years. Yet, in spring individual moose expressed a consistent migration timing across years. There was little synchronization of migration timing between individuals from the same population both in spring and autumn, which may have been related to mild weather conditions. We observed that moose postponed their migrations and started movement toward summer ranges at a similar time window in years when spring was delayed due to harsh weather. Hence, in light of global warming, we presume further changes in animal movements will arise.

## Introduction

Animal movement and space use strategies take diverse patterns ranging from stationary behaviours to seasonal migrations. Migration is a widespread phenomenon across different animal taxa [1]. The most conspicuous are long distance seasonal movements of birds [2], turtles [3], or savanna herbivores [4]. Migration predominantly occurs in spatio-temporally heterogeneous environments and allows animals to adapt to changing resource availability [5–8]. However, most often only a fraction of the population migrates (partial migration), while the remainder stays stationary or nomadic [9–11]. Partial migration is driven by a broad set of interacting factors including predation risk and competition for food resources or breeding territories [7, 12]. At the individual level the migratory tendency can be facultative and change over time, depending on animal intrinsic and extrinsic states (age, body mass, dominance) modified by population density [12–15].

In temperate and arctic climatic zones of the northern hemisphere, large herbivores face spatial and temporal (seasonal) changes in food quantity and quality induced by temperature, precipitation, and snow cover [16–19]. To maximize individual fitness, many ungulate species track the changes in food resources, which often leads to migration and spatially distinct summer and winter home ranges (e.g. red deer *Cervus elaphus* [20], roe deer *Capreolus capreolus* [21], wild mountain reindeer *Rangifer tarandus* [22], moose *Alces alces* [23], woodland caribou *Rangifer tarandus caribou* [24]). Migration distance is a function of changes in the scale of resource distribution in the landscape shaped by snow depth, vegetation productivity and human impact. Hence, ungulates occurring in landscapes that are characterized by large-scale seasonal changes in the resource availability take long-distance migrations, while fine-scale changes lead to short-distance movements [18, 23, 25–27]. In spring ungulates start migration after snow disappearance and track vegetation green-up when moving to summer ranges [5, 28–30]. In autumn, migration is slower than in spring, while migration timing is less synchronized and coincides with the vegetation senescence, snow accumulation, and the onset of hunting season [31–33]. However, spring and autumn migration timing and duration are predicted to alter due to changes in vegetation phenology caused by the future global warming [34, 35].

In moose, the propensity of individuals to migrate and migratory parameters (migration distance, timing and duration) differs significantly between populations and within individuals across years. On the basis of multi-annual movement data from moose populations spanning across the entire Scandinavian Peninsula, Singh et al. [18] demonstrated that the share of migratory individuals within populations decreased southward, which correlated with declining predictability of variation in the resource availability across landscapes expressed as snow depth. The latitudinal differences also applied to migratory parameters, with northern populations characterized by longer migration distances and durations, as well as delayed spring migration and premature return in the autumn [18].

As most of the studies on moose movement behaviours in Europe have been conducted on Scandinavian populations, little is known about the movement strategies of moose at the southern edge of the species’ range. In Eastern Poland, moose actively seek pine-dominated (*Pinus sylvestris*) coniferous forests in winter and wetlands overgrown with willow (*Salix* sp.) in summer [36–38]. The landscape characterized by high habitat patchiness provides moose with a mosaic of small patches of summer (wetlands) and winter (forests) habitats, while low habitat patchiness can lead to a distinct spatial separation of summer and winter habitats and thus may determine moose space use. Therefore, in this study we aimed at exploring the link between habitat patchiness and moose space use in two populations in Eastern Poland at both landscape and individual level. We expected that decreasing habitat patchiness in central Europe would be associated with migratory behaviours (Prediction 1; P1).

At landscape scale (ca. 1000 km^2^), this should result in greater share of migratory individuals in populations inhabiting landscape with lower patchiness of habitats than in populations occurring in landscape where habitats occur in larger patches. At the individual level, in turn, the effect of landscape patchiness on the probability of migration would be expressed in the homogeneity of habitats within moose seasonal ranges. We expected that decreasing habitat patchiness would lead to increasing habitat homogeneity within seasonal ranges i.e. predominance of wetlands in summer range and forests in winter range. Moose would migrate, therefore, when within their summer home range, there was not enough forest habitats that provided forage supply to survive winter. To verify this hypothesis, we tested an association between moose propensity to migrate and the abundance of forests within their summer ranges.

As a second aim of this study, for those individuals that expressed migratory behaviour, we investigated moose migratory parameters (i.e., migration distance and duration, migration timing and time spent on summer range) and tested a set of predictions (P2-P4) to explain the variation in individual movements. We expected the spring migrations would be quicker that autumn returns (P2). We expected this because moose in spring would migrate to summer ranges to quickly restore body reserves after the period of prolonged food shortage, whilst in autumn moose would much more slowly return to generally poorer wintering habitats. Secondly, due to more rapid temporal changes in vegetation quality in spring than in autumn (green-up vs senescence) [39], we predicted stronger synchronization of migration timing among individuals from the same population in spring (P3). The time of spring migrations, in turn, should be associated with the weather severity, i.e., harsh weather conditions at the end of winter and the beginning of spring would postpone migrations due to late vegetation green-up (P4). Since in Eastern Poland severe weather conditions in autumn have become very rare due to climate change in the recent decade, we did not expect their effect on movement in this season.

## Material and methods

### Study area and sites

**Fig 1:**
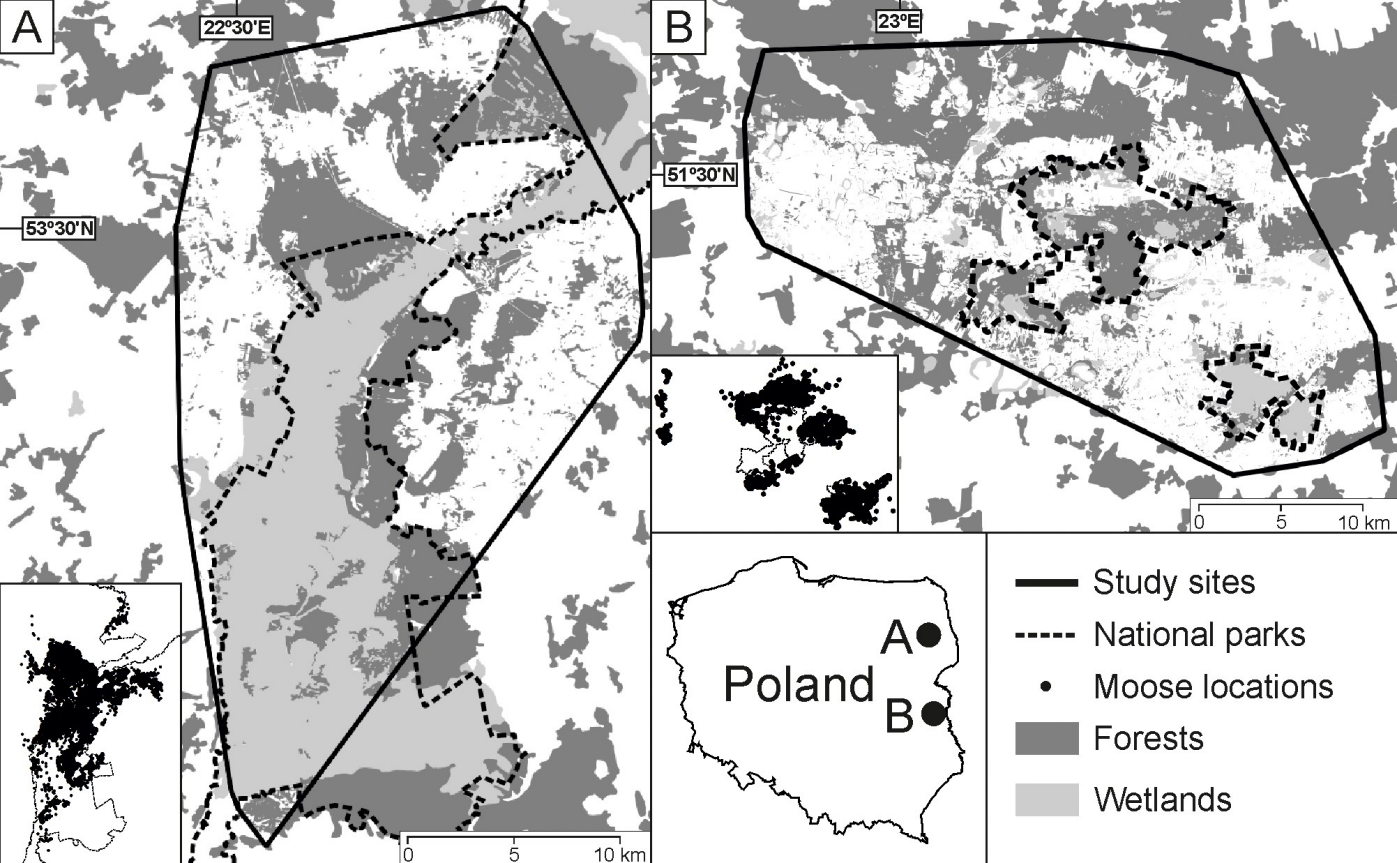
Distribution of the Biebrza (A) and Polesie (B) study sites in eastern Poland during telemetric research in 2012–2017. Study sites were delineated as minimum convex polygons (MCP 100%).

Our study area covers two refugial areas of moose in eastern Poland (Fig 1).

The northern study site (hereafter Biebrza: 22°35’E, 53°26’N) spans across the southern part of Biebrza National Park and surrounding forest districts. The southern part of Biebrza NP protects a valley of the Biebrza river, which is the largest marshland in Central Europe. Biebrza valley is a longitudinal peat basin, 12–15 km wide, surrounded from the East and West by glacial uplands up to 10–20 m high [40]. Vegetation on wetlands is dominated by sedge, sedge-moss, and reed communities. Forest communities consist of black alder *Alnus glutinosa*, downy birch *Betula pubescens*, and coniferous bog forests [41]. Elevated areas at the border and outside Biebrza valley are covered by coniferous and mixed-coniferous forests [42].

The southern study site (hereafter Polesie: 23°8’E, 51°26’N) lays in the West Polesie Biosphere Reserve, which covers Polesie National Park and its buffer zone. Polesie NP is covered by plains of organic origin and characterized by high spatial heterogeneity of habitats [43]. Patches of fen habitats are interwoven with typical forest habitats and small water bodies that are overgrown with organic material. Bogs are overgrown with fen plant communities [44]. Forest communities are dominated by black alder, downy birch, and coniferous bog forests [45]. In both study sites, four ungulate species–moose, red deer, roe deer, and wild boar *Sus scrofa–*are present. The most abundant of these are roe deer [46]. All ungulate species are prey species of the wolf *Canis lupus* [47].

The climate of both study sites is transitional between Atlantic and continental types. In Biebrza, winter (period with the average daily temperature below 0°C) is long (107–117 days) whereas summer (period with the average daily temperature above 15°C) is short (77–85 days). Growing season lasts 190 days, on average [48]. Snow cover persists up to 110–120 days. Mean maximum snow cover is 30 cm. The warmest month is July (mean = 17.5°C), the coldest is February (mean = -5°C). Annual precipitation is 550 mm. In the Polesie both summer and winter are long– 105 and 110 days, respectively. Growing season lasts 211 days, on average. Mean maximum snow cover is 25 cm, and persists 60–70 days, on average. The highest mean monthly temperature is in July (mean = 17.9°C), whilst the lowest in January (mean = -3.9°C). Annual precipitation is about 550 mm and summer rains represented 40% of the annual precipitation [49]. Weather data used in the analyses were collected by the Institute of Meteorology and Water Management, National Research Institute, Poland from the weather stations in Kopytkowo (Biebrza) and Włodawa (Polesie).

### Movement data

In movement analyses, we used GPS locations of adult moose (≥ 2 years) collected in Biebrza (2012–2017; 12 females and 10 males) and Polesie study sites (2013–2018; 9 females and 1 male). This study was carried out under the written research permits (Biebrza: no. DL.gł-6713-6/5531/11/abr, DLPgł-6713-4/4478/13/RN, DOPpn-4102-975/50129/10/RS, DLPpn-4102-91/8170/13/KW; Polesie: DL-gł-6713-47/50885/11/abr, DLPgł-6713-6/2403/13/RN, DLPgł-6713-6/15699/13/RN, DLP-VIII-6713-25/35939/14/RN, DLP-VIII.6713.8.2016.ABR, DOPpn-4102-834-40251/11/RS, DLPpn-4102-34/2130/13/RS, DLP-III-4102-65/4900/14/MD, DLP-III-4102-449/32714/14/MD, DLP-III-286-10-2015/MD) from the Polish Ministry of Environment. This study was approved by the State Ethics Committee for Animal Experimentation in Białystok (no. 10/2010, 57/2011) and Lublin (no. 15/2011, 2/2013, 92/2015), Poland. Animals were immobilized from a car with a dart gun (Dan-Inject) and etorphine [50], and then fitted with Ecotone Telemetry GPS-GSM collars. The study was conducted on public land. We confirm that no endangered or threatened species or locations were involved in this study. During the study we did not observe any negative impact of the animal collaring on individual behaviour and survival. At the end of the study 38% of individuals were alive, remaining moose either died due to different causes (starvation, poaching, road accidents) or their destiny was unknown (collar failures).

### Data analyses

#### Habitat patchiness and winter severity data

We described the habitat patchiness of the two study sites as the density and mean size of patches of the two most important habitats–forests and wetlands [36]. Spatial outlines of study sites were defined by fitting minimum convex polygons (MCP 100%) to all moose positions. On the basis of vector habitat map (an accuracy of 0.001 km^2^; Database of Topographic Objects) acquired from Head Office of Land Surveying and Cartography, using the ArcView GIS software by ESRI (version 9.3.1), we calculated the density (number of patches per 100 km^2^) and mean size of forest and wetland patches in the two study sites. Furthermore, for the Biebrza study site, we collected data on snow cover and mean daily temperature to link them with migration parameters.

#### Movement classification

We applied the net squared displacement (NSD) method to classify moose behaviour patterns [51–53]. NSD provides squared straight-line distance between starting position of an individual within its starting range and every subsequent position and has been commonly used for classification of animals movements basing on telemetry data [18, 20, 22, 54]. Firstly, prior to NSD analyses, we visually scanned for outliers and extracted one location per day (first position acquired in a given day) [52]. Then, we divided these locations into yearly subsets (Biebrza: 40 yearly subsets for females and 18 for males; Polesie: 20 and 4, respectively), which started on the 15th of February each year–time preceding moose migration into summer ranges. We retained only those subsets which spanned at least 335 days. Finally, for each yearly data subset, we used ‘findrloc’ and ‘mvmtClass’ functions from “MigrateR” package [53] to: (1) test different starting positions (from 15th February to 2nd March) and for each of them calculate NSD, (2) fit five different non-linear models, each representing different movement behaviour (migration, mixed migration, dispersion, nomadism, and residency; S1 Fig) to the NSD calculated for every starting position, (3) select the best starting position and best movement strategy on the basis of Akaike information criterion (model with the lowest AIC) [52, 55].

Mysterud et al. [20] and Singh et al. [56] highlighted the problem of possible misclassification when using an automatic movement classification, especially when surveyed individuals migrated at short distances. Thus, we visually inspected all plots presenting temporal movement patterns (NSD plots) to evaluate classification correctness. After visual examination, we altered part of movement classifications (S1 Table). We consider an individual as: 1) migrant–when after winter moose had migrated into summer range and then returned to the same winter range, 2) mixed migrant–when after winter moose had moved into summer range and then migrated to winter range which did not spatially overlap the winter range occupied in a previous year, 3) disperser–when after winter moose had moved and then established a new spatially separate range which occupied during the next winter, 4) nomadic–when moose moved in an unpredictable way throughout year and individual established neither winter nor summer ranges, and (5) resident–when moose did not move outside winter range, i.e., winter and summer home ranges spatially overlapped. For migratory and mixed migratory individuals, we applied additional assumption on a minimum time of summer range occupancy. An animal was classified as either migratory or mixed migratory when it spent at least 21 days on spatially separate summer range [54, 57]. Furthermore, we added sixth category termed “ambiguous movements”, i.e., all movement patterns that did not fulfil the above-mentioned assumptions.

#### Migration parameters

On the basis of the results of movement classification, we calculated the percentage of individual movement categories separately in each study site. Then for individuals from Biebrza classified as migratory (N = 38), on the basis of modelling results computed with “MigrateR” package, we described the following migration parameters: migration distance, timing of spring and autumn migrations (start and end of migratory movements), duration of migration, and time spent on summer range [53]. Before we calculated population averages, we averaged migration parameters over years for moose that were tracked for two or more years.

#### Statistical analyses

Firstly, we tested statistical differences in the share of migrant and stationary moose between Polesie and Biebrza study areas with Fisher’s exact test. The difference in mean habitat patch size between study areas was investigated with Kruskal-Wallis test (P1).

Secondly, to test if availability of forests in moose summer home ranges affects moose movement strategy, we applied a generalized additive model (GAM) for binomial data in the *mgcv* package implemented in R (GAM1; [58]). For this purpose, we pooled migratory and mixed-migratory individuals into a common migratory class and set movement strategy (migratory vs stationary) as a dependent binomial variable while forest within summer home ranges as single explanatory variable. For each migratory (combined migratory and mixed-migratory movement class) and stationary individual and each year, we delineated summer home ranges by fitting Brownian Bridge Movement model 95% (BBMM 95) (N = 64; median = 5.2 km^2^; range = 1–40.4 km^2^; [59]) to summer moose locations (24 positions per day). We did not include ambiguous and dispersing individuals in analyses. For migratory individuals, summer was a period indicated as summer range by NSD method. For sedentary individuals, we described summer range as a period between median date of departure of migratory moose from winter to summer ranges (22th of April) and median date of return movements (17th of October). Then, we intersected BBMMs with a vector habitat map (0.001 km^2^) and summarized the area of forest polygons (all forest types) within every summer home range (median forest area = 1.6 km^2^; range = 0.0004–25.3 km^2^).

For Biebrza population, we tested the difference in duration of spring and autumn migration with Kruskal-Wallis test (P2). We checked the differences in synchronization of migration timing in spring and autumn by testing the equality of variances for departure time with Levene’s test (P3). Because we found a high variation in migration timing in both seasons, we investigated the individual consistency in departure time across years. Under GAM framework, we tested if departure time in preceding year (independent variable) predicted the start of migration in a given year (dependent variable, spring: GAM2; autumn: GAM3). In addition, with GAM we surveyed association between the time of spring departures to summer ranges (independent variable) and the time of autumn returns to winter ranges (dependent variable; GAM4). We sampled some individuals over multiple years, therefore, we added a year of investigation and individual identification number (ID) as two random factors to all GAM models (GAM1-GAM4) as penalized regression terms [58]. For all four GAMs, we compared different compositions of random terms (i.e., Year vs ID, vs Year + ID) with AIC. The best models were models with the lowest AIC values (S3 Table).

Finally, we fitted two generalized additive quantile models to test the effect of mean daily temperature in spring (independent variable; February 15 –April 15) and autumn (October 15 –December 15) on the start of migrations in both seasons (dependent variable) at 0.1, 0.25, 0.5, 0.75, 0.9 quantiles (spring: qGAMs_spring_, autumn: qGAMs_autumn_; [58]) (P4). In both qGAMs, we set individual ID as a random factor. All movement and statistical analyses were made in R [60].

## Results

### Habitat patchiness and its effect on movement strategy

Biebrza study site had significantly higher share of migratory individuals and a lower patchiness of both forest and wetland habitats compared to the Polesie study site (Fisher’s exact test, *P* < 0.001) (P1). In Biebrza, the density of forest patches was 177.9/100 km^2^, while in Polesie it was 302.5 patches/100 km^2^. This discrepancy also confirmed mean patch size which was significantly higher in Biebrza than in Polesie (0.17 and 0.13 km^2^, respectively; Kruskal-Wallis test, *Χ*^2^ = 134.7, P < 0.001). The differences in habitat patchiness between study sites were even larger when looking at wetland habitats. The density of wetland patches was almost 9 times higher in Polesie than in Biebrza (360.3 and 41.3 patches/100 km^2^, respectively), while mean size of wetland patches was significantly greater in Biebrza (0.71 km^2^) than in Polesie (0.02 km^2^; Kruskal-Wallis test, *Χ*^2^ = 68.2, P < 0.001).

**Fig 2:**
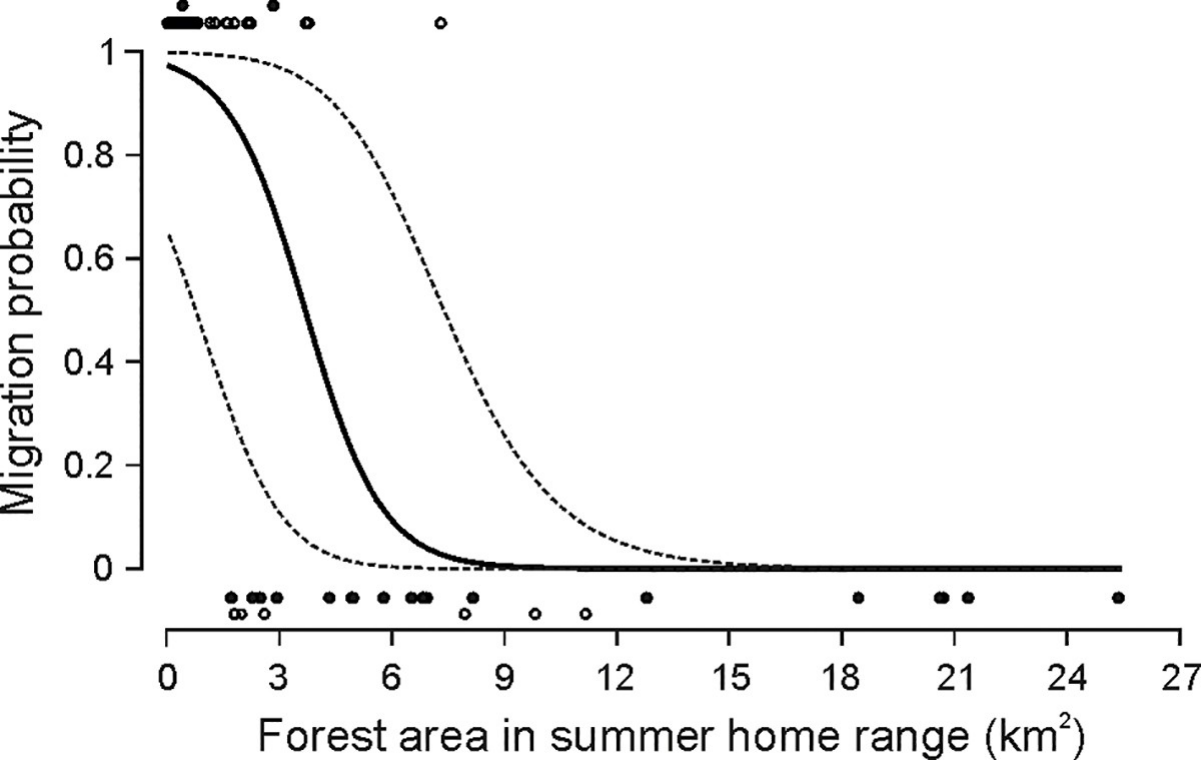
The predicted propensity of individual moose to migrate in relation to forest area in summer home range (Brownian Bridge Movement model 95%; GAM1). Dots at the top and the bottom of the chart represent summer home ranges of individual moose which in a given year was assigned either to migratory (top) or stationary (bottom) class. Open dots are summer home ranges from Biebrza study site, black dots–home ranges from Polesie study site.

The GAM1 indicated a significant negative association between the abundance of forest in moose summer home ranges and the propensity of moose to migrate (slope = -0.98 ± 0.46, *Z* = -2.11, *P* = 0.034). With increasing forest area in summer home ranges from 0 to 8 km^2^, the probability of individual moose migration decreased from 97.2 to 1.5% (Fig 2).

### Movement classification and migration parameters

In Biebrza, 65% of individuals were classified as migratory, 16%–resident, and 19%–ambiguous. The percentage of switches between movement behaviours in two consecutive years for an individual moose tracked for at least two years (N = 12) was 22%. The majority of switches (62%) occurred between ambiguous and migratory or resident movement strategies (S2 Table). In Polesie, movement classification was as follows: 4%–migration, 4%–mix-migration, 79%–residence, 4%–dispersal, and 9%–ambiguous behaviour (S2 Table). In this study site, we indicated two switches between movement strategies–from ambiguous movement to residence and from dispersal to migration. All migratory females, except for one individual in Biebrza, took single migrations between winter and summer ranges. The exceptional female displayed a double migration. Each year, this particular female, beside typical spring and autumn migrations, migrated also for a few weeks to winter range in summer (July-August).

**Fig 3:**
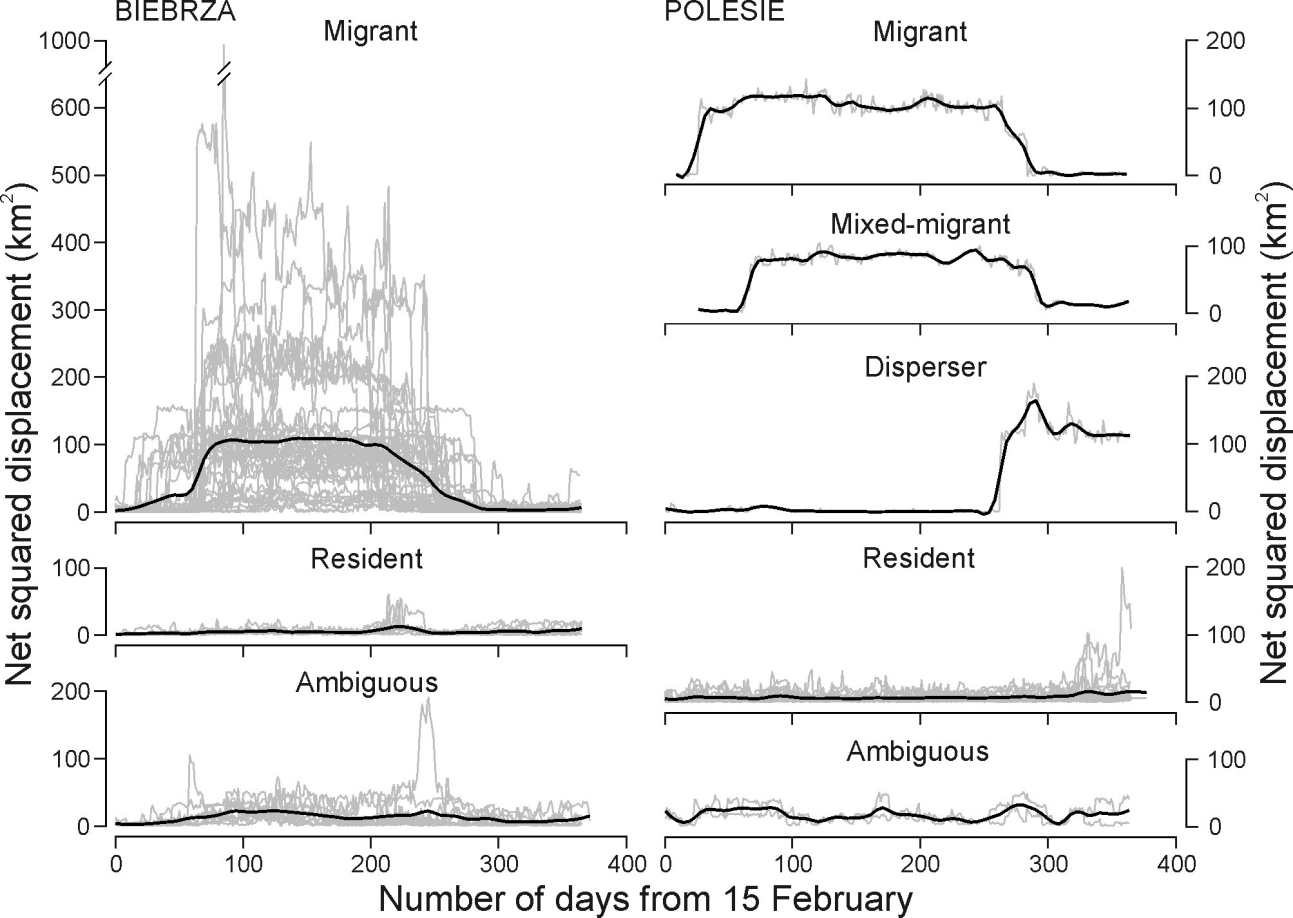
Moose movement strategies in Biebrza and Polesie classified on the basis of the net squared displacement (NSD) method. Each grey line represents an individual moose-year trajectory (Biebrza: N = 58 moose-years, Polesie: N = 24 moose-years). Black lines show the local fitting of polynomial regression (loess) to NSD data.

In Biebrza, moose migrated 9.2 km on average (SD = 4.7 km, range 2.9–20.1 km; Fig 3).

**Fig 4:**
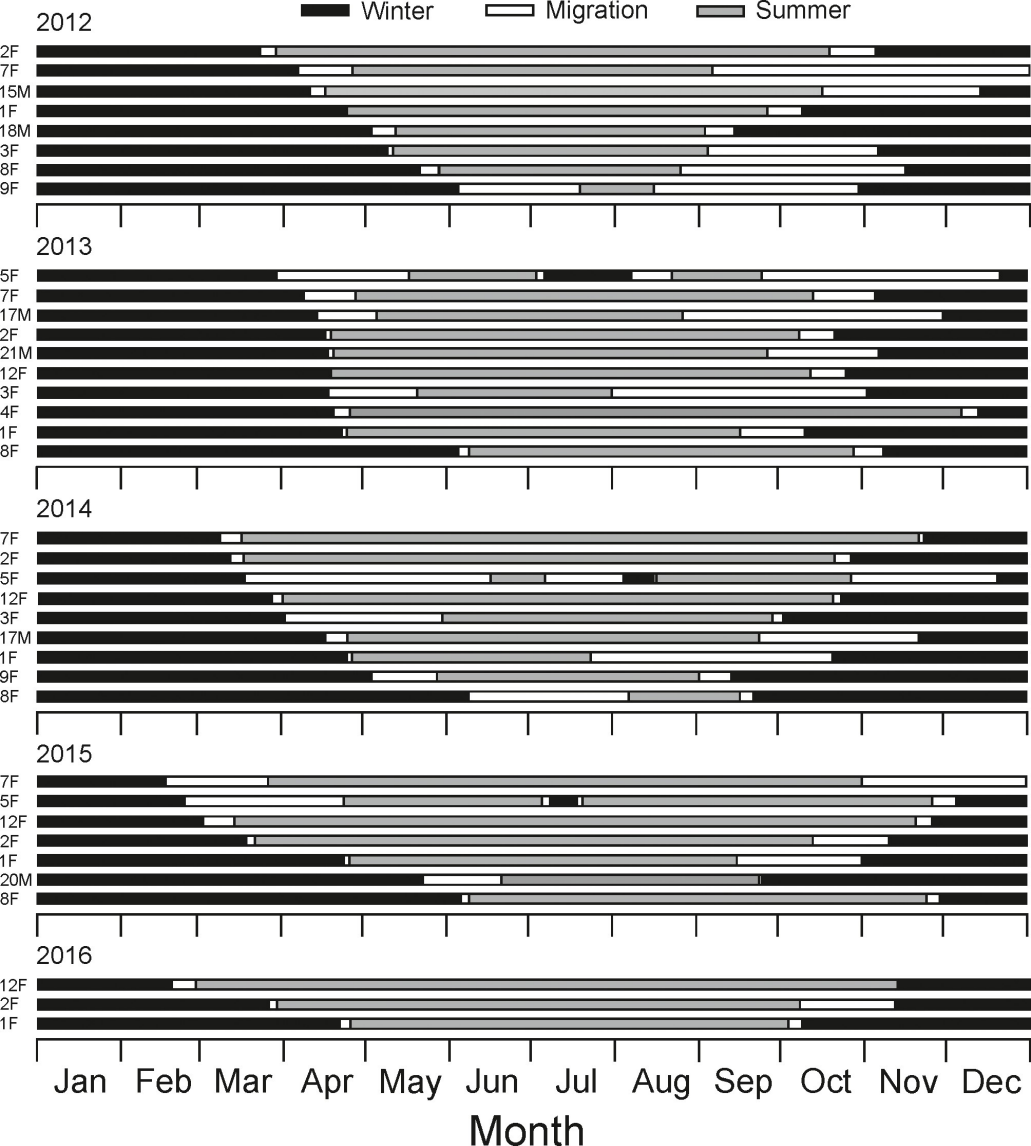
Duration and timing of migratory movement of moose in Biebrza. Migration parameters derived from non-linear models fitted to NSD data. Female 5F migrated twice between winter and summer home ranges.

For individuals that were tracked longer than one year, the standard deviation (SD) of migration distances across years varied from 0.6 to 1.7 km. Although the migration distance was relatively short, moose spent variable amounts of time moving between seasonal ranges, and the duration of moose migration in spring (mean ± SD = 15.9 ± 18.0 days, range 1–68 days) was significantly shorter than in autumn (mean ± SD = 38.4 ± 21.2 days, range 1–118 days; Kruskal-Wallis test, *Χ*^*2*^ = 5.93, *P* = 0.01) (P2). Moreover, each individual spent variable amounts of time on spring and autumn migration throughout tracking years (spring: SD ranged from 1 to 23.5 days, autumn: from 4.3 to 43.9 days, Fig 4).

Moose occupied their summer range for 137.7 days, on average (SD = 50.7 days, range 26–258 days). Moose individuals varied in the amount of time spent on summer ranges across surveyed years (SD ranged from 7.3 to 45.1 days; Fig 3).

**Fig 5:**
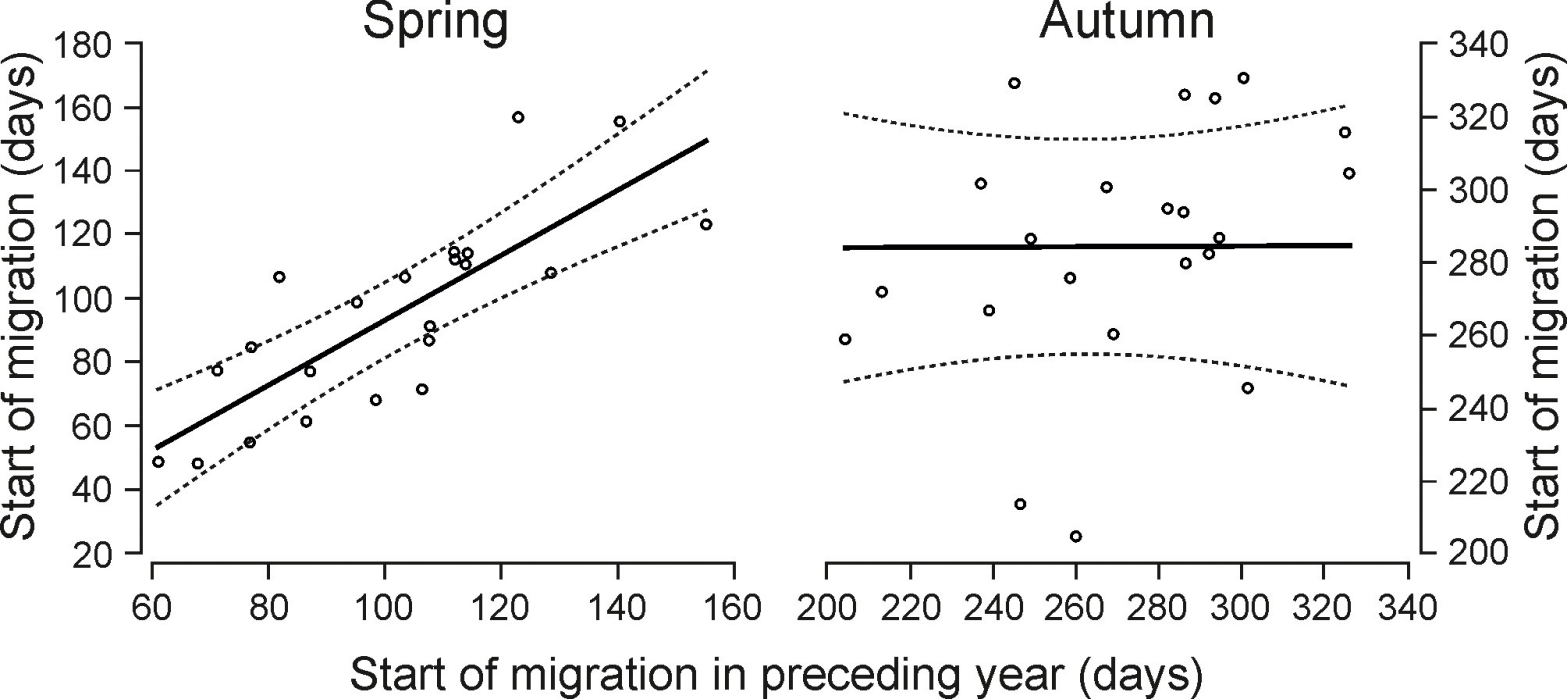
The start of moose migrations predicted by the time of departures in preceding year in Biebrza (GAM2, GAM3).

In the spring, moose started their migration on the 19th of April (SD = 25.3 days, range: February 17 –June 9) and ended on 9th of May (SD = 36.3 days, range: February 27 –August 7) on average. In the autumn (return movement), the average migration start occurred on the 3rd of October (SD = 27.4 days, range: July 24 –December 7), while the average migration end on 7th of November (SD = 29.3 days, range: September 13 –December 31). The synchronization of migratory movements among individuals did not differ between spring and autumn–the time of migration start in spring and autumn was equally variable (Levene’s test; *F*-value = 0.25, *P* = 0.62) (P3). Nevertheless, in spring 2013 occurred remarkably greater synchronization of movements–the standard deviation of migration initiations in 2013 (SD = 16.7 days) was one and a half times as low as in 2012 and 2014 (23.1 and 27.8 days, respectively), even though a similar number of animals was tracked. There was lower individual variability in the time of beginning and end of migratory movements in spring than in autumn across years (start: spring–SD range = 1.4–22.9 days, autumn–SD range = 5.7–38.4 days; end: spring–SD range = 0.7–19 days, end–SD range = 4.6–29.3 days; Fig 4). Unlike in the autumn, spring departure time was significantly positively correlated with the date of migration initiation in preceding year (spring: GAM2, slope = 1.02 ± 0.17, *Z* = 5.89, *P* < 0.001; autumn: GAM3, slope = 0.005 ± 0.23, *Z* = -2.10, *P* = 0.98, Fig 5).

**Fig 6:**
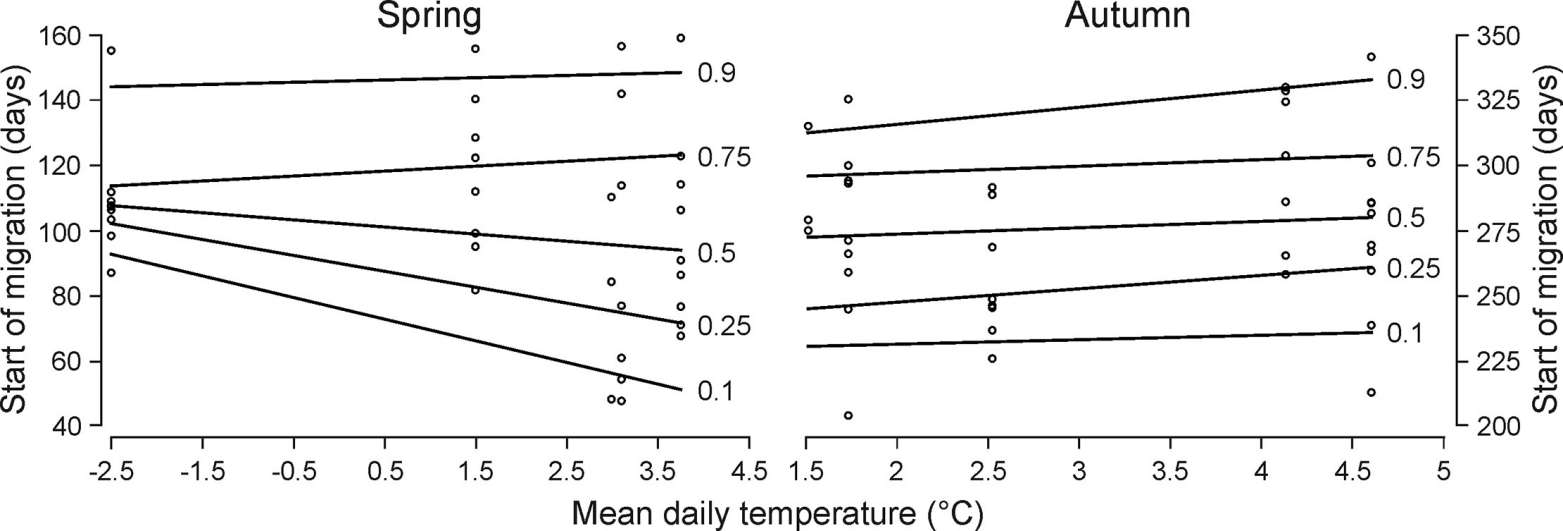
The predicted effect of mean daily temperature in spring (February 15 –April 15) and autumn (October 15 –December 15) on the start of moose migrations in both seasons in Biebrza. Results of the generalized additive quantile regression models (qGAMs_spring_, qGAMs_autumn_).

The time of spring departures was negatively associated with the time of moose returns to winter ranges (GAM4, slope = -0.46 ± 0.15, *Z* = -3.05, *P* = 0.004; S2 Fig). Quantile regression indicated that decreasing temperature in early spring was significantly positively associated with the start of migratory movement of individuals that migrated earlier than median date of departures to summer ranges in the population (qGAMs_spring_, 0.1 quantile: *P* = 0.001, 0.25 quantile: *P* = 0.008; S4 Table) (P4). At 0.1 quantile, 6°C lower temperature in spring postponed the start of migration by 40 days (Fig 6).

We did not indicate such a relationship for autumn migrations (qGAMs_autumn_; S4 Table; S3 Fig).

## Discussion

In Europe, space use and migratory behaviour of moose have so far been studied mainly in the northern latitudes in Scandinavia [17, 18, 23, 52]. We expected that in the southern-most populations of the species in Europe, the proportion of different movement strategies would be predominantly driven by the level of habitat patchiness. Previous studies have shown that animal movement strategies can result from spatio-temporal differences in availability of food resources [20, 23, 26]. The scale of movements was a function of environmental diversity–the larger the scale of seasonal variation in a landscape, the higher the proportion of animals seasonally migrating and the greater the migration distances [18, 23]. Movement behaviours were closely associated with latitudinal changes in the predictability of resource distribution in the landscapes. Therefore, migratory behaviours in northern latitudes prevailed, while southern populations had a higher share of other movement types: nomadic, resident, and disperser [18].

Yet, we demonstrated that in southern latitudes there can be moose populations dominated by individuals expressing different movement strategies. In Biebrza, the majority of moose migrated, while in Polesie moose remained resident. We expected this difference to stem from habitat patchiness which was lower in Biebrza compared to Polesie (P1). The most apparent differences concerned wetlands providing preferable food resources in summer, which constituted larger patches in Biebrza and numerous small plots in Polesie. In Polesie, wetlands and forests create a more intricate mosaic of habitats, while in Biebrza in many places these habitats are clearly spatially separated [37, 61, 62]. This habitat structure in Biebrza drives spatio-temporal variation in food availability. Seasonal ranges then were spatially separated and dominated by two different habitats–wetland in summer and forest in winter.

From the perspective of individual moose, migration took place when summer range lacked in habitats which could have provided forage supply in winter (coniferous forests). Thus, the migration probability decreased with increasing abundance of forests in summer home ranges. Interestingly, among moose classified as ambiguous in Biebrza, there were mostly individuals that moved to the neighbouring marshes in the spring but retained a connection with their winter home range, taking regular and multiple trips to these winter habitats. Such behaviour was a common phenomenon across different ungulates species (roe deer [57], red deer [20], moose [56]). In many cases this raised a problem with the precise distinction of migratory behaviour that led to the development of the “migratory continuum” concept [7, 19]. Gurarie et al. [21] proposed a solution where binary migratory vs. non-migratory framework was replaced by the calculation of a “migratoriness” index describing the degree of seasonal range shifting of an animal.

In Biebrza, at the individual level, we observed that some moose were conservative and took the same movement strategies across years, while others switched their movement behaviour from year to year. The rate of switching was relatively low and similar to that observed in elk population in Canada (15% [54]) or moose in Sweden (9% [63]) and Alaska (USA) (21% [64]). Though switches between movement strategies seem to help animals maximize lifetime reproductive success, there has been little evidence supporting this hypothesis in large herbivores. To date, limited surveys on the switching strategy have revealed that switching probability can be driven by intrinsic, environmental, and density-dependent factors [54]. It seems that the ambiguous class, which consisted mostly of moose with partly overlapping seasonal ranges, was an intermediate stage between the resident and migratory movement strategies. Hence, switches between ambiguous and migratory classes can be treated as the transition along the gradient of “migratoriness” rather than true switches between distinctive movement strategies [21].

In Biebrza, moose took relatively short-distance migrations compared to those observed in the northern part of the species range in Europe and Northern America [18, 31, 52]. As the migration distance was proved to be associated with a spatial scale at which food resource changed, migration distances of moose in Biebrza seemed to be congruent with a relatively small spatial scale at which food supply seasonally changed in the site [23, 26]. Migration distance varied considerably among individuals but displayed similar values within individuals that were tracked for at least two years which can confirm a high fidelity of seasonal ranges [65, 66].

Moose spent variable amounts of time moving between seasonal ranges. In Biebrza, many migratory moose moved at very low rates between seasonal home ranges, which led to gradual and directed enlargement of winter or summer home ranges that eventually caused home range separation. Such behaviour can prove that habitats that moose intersect when moving between seasonal ranges can provide high quality forage. For example, during spring migrations mule deer (*Odocoileus hemionus*) made a series of stopovers to track phonology green-up that elongated the duration of their migrations [67]. In our study, the duration of moose migration in autumn was substantially longer than in the spring (P2). Shorter duration of migration in spring could reflect an animal’s need to reach high quality food resources to restore post-winter body condition deficits. For females, rapid reaching of optimal and safe places for giving birth may be also crucial [68]. On the other hand, this might have been caused by mild weather conditions in autumns and early winters that drove an elongation of migration time and delayed returns to winter ranging areas [31].

In Biebrza, both spring and autumn migration timing expressed large between-individual variation and spanned over four months. Contrary to our expectation (P3), moose migrations were equally out of sync in both seasons. Only in springs with severe weather conditions, individuals known for having early migration starting in February postponed their departures to summer ranges (P4) that caused remarkably higher movement synchronization. Previous surveys proved that in spring animals synchronized their movements to follow vegetation green-up and maximize energy intake (forage-maturation hypothesis) [28–30]. In our study area, this can be especially true for springs with severe weather conditions, when a high rate of vegetation green-up can be expected in the aftermath [69, 70]. In mild springs, the process of vegetation greening could stretch over much longer period that might cause diversified reactions of moose individuals to on-going changes in surrounding environment. We observed, therefore, individuals starting departures to summer ranges as early as the end of February. It is worth stressing that individuals which migrated in early spring returned to their winter home ranges later than those that started their movements in the late spring or early summer. This can imply that quicker reaction to the first stages of vegetation greening expressed individuals that minimized time spent in winter ranges. Interestingly, individuals departed to summer ranges at a similar time window across years that can suggest that the decision on migration may involve some kind of inherited or gained knowledge on the expected conditions at their summer ranges at different time of the year.

Although Ball et al. [17] indicated that moose started synchronized migration to winter ranges shortly after the appearance of heavy snowfall, we did not find an association between autumn weather conditions and migration timing of moose. During the study we did not register severe weather conditions in autumn and early winter. The periods with continuous snow cover and frosts occurred as early as the end of December when all individuals were already back in winter ranges. The lack of response to weather conditions in autumn was also observed by Rivrud et al. [32] and Debeffe et al. [33] in red deer (Norway), that migrated to winter ranges well before snow fall and frost. Therefore, it seems that autumn migrations in Biebrza could have been triggered by other factors such as vegetation senescence which might express a high interannual variation. Finally, the migration propensity and the timing of seasonal movements of moose can be sex-specific [18]. Unfortunately, due to much higher mortality of males due to different factors (starvation, poaching, traffic accidents), we were not able to collect enough data on male migration to make reliable inferences. Nonetheless, excluding male data from analyses did not change the direction of relationships nor the significances of the obtained results.

To sum up, with our study we confirmed that at the south-western edge of the species’ range, moose movement strategies can express substantial variation as a result of the diverse level of habitat patchiness. Global warming and mild winters seem to exert profound effects on migratory parameters, and may therefore alter animal space use and behaviour as well as introduce less synchronism and predictability in observed patterns. Thus, further studies on the effects of climate change on animal behaviour are highly important to understand the problem and elaborate adequate management or protection strategies [35, 71].

## Supporting information

S1 Table Collation of moose movement classification obtained through automatic procedure (“Migrate R” package), with classification corrected by authors after visual examination of non-linear models from automatic classification. (DOCX)

S2 Table Classification of moose movements in Biebrza and Polesie study sites during 2012–2017 on the basis of the net squared displacement method. (DOCX)

S3 Table Model selection (based on the AIC criteria) for optimal random term structure in the considered GAMs. (DOCX)

S4 Table Results of additive quantile regressions (qGAM1, qGAM2) for the effect of mean daily temperature in spring (February 15—April 15) and autumn (October 15 –December 15) on the start date of moose migrations in Biebrza study area. (DOCX)

S1 Fig Conceptual visualization of the five non-linear models fitted to net squared displacement (NSD) data. (DOCX)

S2 Fig The predicted association between the time of departures of moose to summer ranges and the time of their returns to winter ranges in Biebrza. (DOCX)

S3 Fig The collation of weather data (snow cover and mean daily temperature) with the data on the start of migratory movement of moose in Biebrza. (DOCX)
