# Supplementary material for: The level of habitat patchiness influences movement strategy of moose in Eastern Poland
Source: PLoS One. 2020 Mar 19;15(3):e0230521. doi: 10.1371/journal.pone.0230521 (PMC7082038; doi:10.1371/journal.pone.0230521)
Supplement: S1 Table — (DOCX) [file pone.0230521.s001.docx]

S1 Table. Collation of moose movement classification obtained through automatic procedure (“Migrate R” package), with classification corrected by authors after visual examination of non-linear models from automatic classification. For more details see Materials and Methods. B – Biebrza study site, P – Polesie study site.

|  |  | Modified classification | | | | | | | | | |
| --- | --- | --- | --- | --- | --- | --- | --- | --- | --- | --- | --- |
|  |  | Migrant | | Mixed migrant | | Resident | | Disperser | | Ambiguous | |
|  |  | B | P | B | P | B | P | B | P | B | P |
| Automatic classification | Migrant | 23 | 1 | - | - | 1 | - | - | - | - | 1 |
|  | Mixed migrant | 15 | - | - | 1 | 6 | 13 | - | 1 | 12 | - |
|  | Resident | - | - | - | - | - | 4 | - | - | - | - |
|  | Disperser | - | - | - | - | 1 | - | - | - | - | 1 |
|  | Nomad | - | - | - | - | - | 2 | - | - | - | - |
