# Supplementary material for: The level of habitat patchiness influences movement strategy of moose in Eastern Poland
Source: PLoS One. 2020 Mar 19;15(3):e0230521. doi: 10.1371/journal.pone.0230521 (PMC7082038; doi:10.1371/journal.pone.0230521)
Supplement: S2 Table — (DOCX) [file pone.0230521.s002.docx]

S2 Table. Classification of moose movements in Biebrza and Polesie study sites during 2012-2017 on the basis of the net squared displacement method (see Materials and Methods). Moose movement strategies: MI – migrant, MIX-MI – mixed migrant, DI – disperser, RE – resident, AM – ambiguous.

| ID | Sex | Movement classification | | | | | |
| --- | --- | --- | --- | --- | --- | --- | --- |
|  |  | 2012 | 2013 | 2014 | 2015 | 2016 | 2017 |
| BIEBRZA | | | | | | | |
| 1 | F | MI | MI | MI | MI | MI | - |
| 2 | F | MI | MI | MI | MI | MI | - |
| 3 | F | MI | MI | MI | - | - | - |
| 4 | F | AM | MI | RE | - | - | - |
| 5 | F | RE | MI | MI | MI | AM | - |
| 6 | F | RE | - | - | - | - | - |
| 7 | F | MI | MI | MI | MI | AM | - |
| 8 | F | MI | MI | MI | MI | - | - |
| 9 | F | MI | MI | MI | - | - | - |
| 10 | F | - | RE | - | - | - | - |
| 11 | F | - | AM | - | - | - | - |
| 12 | F | - | MI | MI | MI | MI | - |
| 13 | M | RE | - | - | - | - | - |
| 14 | M | AM | - | - | - | - | - |
| 15 | M | MI | - | - | - | - | - |
| 16 | M | RE | - | - | - | - | - |
| 17 | M | AM | MI | MI | - | - | - |
| 18 | M | MI | - | - | - | - | - |
| 19 | M | RE | - | - | - | - | - |
| 20 | M | RE | AM | AM | MI | - | - |
| 21 | M | - | MI | - | - | - | - |
| 22 | M | - | AM | AM | AM | AM | - |
| POLESIE | | | | | | | |
| 1 | F | - | MIX-MI | - | - | - | - |
| 2 | F | - | - | RE | - | - | - |
| 3 | F | - | - | RE | RE | RE | RE |
| 4 | F | - | - | RE | - | - | - |
| 5 | F | - | - | AM | AM | RE | RE |
| 6 | F | - | - | - | RE | RE | - |
| 7 | F | - | - | - | RE | RE | RE |
| 8 | F | - | - | - | - | DI | MI |
| 9 | F | - | - | - | - | RE | RE |
| 10 | M | - | - | RE | RE | RE | RE |
