# Supplementary material for: The level of habitat patchiness influences movement strategy of moose in Eastern Poland
Source: PLoS One. 2020 Mar 19;15(3):e0230521. doi: 10.1371/journal.pone.0230521 (PMC7082038; doi:10.1371/journal.pone.0230521)
Supplement: S3 Table — (DOCX) [file pone.0230521.s003.docx]

S3 Table. Model selection (based on the AIC criteria) for optimal random term structure in the considered GAMs. GAM1 investigated the propensity of individual moose to migrate in relation to forest area in summer home range (Forest). GAM2 and GAM3 aimed at assessing the effect of time of moose migration initiations in preceding year (D_Time-1_) on the start of migrations in a given year (D_Time_) in spring and autumn (respectively). GAM4 tested association between the time of departures of moose to summer ranges (D_Time_) and the time of returns to winter ranges (R_Time_). AIC – Akaike’s Information Criterion; ΔAIC – difference in AIC between the specific model and the most parsimonious model. Individual identification number (ID) and year were set as random factors.

| Model | AIC | ΔAIC | Model | AIC | ΔAIC |
| --- | --- | --- | --- | --- | --- |
| **GAM1** |  |  | **GAM2** |  |  |
| Forest + ID | 35.45 | 0 | D _Time-1_ + ID + Year | 194.0 | 0 |
| Forest + ID + Year | 35.45 | 0 | D _Time-1_ + Year | 194.1 | 0.10 |
| Forest + Year | 45.34 | 9.89 | D _Time-1_ + ID | 195.6 | 1.56 |
| Forest | 45.34 | 9.89 | D _Time-1_ | 195.7 | 1.75 |
| Intercept | 87.63 | 52.2 | Intercept | 215.4 | 21.5 |
| **GAM3** |  |  | **GAM4** |  |  |
| D _Time-1_ + ID + Year | 214.2 | 0 | D_Time_ + Year | 351.2 | 0 |
| D _Time-1_ + ID | 215.5 | 1.32 | D_Time_ + ID + Year | 351.5 | 0.22 |
| D _Time-1_ + Year | 219.3 | 5.12 | D_Time_ + ID | 351.5 | 0.23 |
| D _Time-1_ | 219.5 | 5.30 | D_Time_ | 351.5 | 0.23 |
| Intercept | 220.7 | 6.58 | Intercept | 357.7 | 6.42 |
