# Supplementary material for: The level of habitat patchiness influences movement strategy of moose in Eastern Poland
Source: PLoS One. 2020 Mar 19;15(3):e0230521. doi: 10.1371/journal.pone.0230521 (PMC7082038; doi:10.1371/journal.pone.0230521)
Supplement: S4 Table — (DOCX) [file pone.0230521.s004.docx]

Table S4. Results of additive quantile regressions (qGAM1, qGAM2) for the effect of mean daily temperature in spring (February 15 - April 15) and autumn (October 15 – December 15) on the start date of moose migrations in Biebrza study area. Temperature was set in the model as a fixed parametric term, while individual identification number (ID) as a random nonparametric (smooth) term. *Edf* and *Χ^2^* described random effect terms.

| Quantile | Estimate | Coefficient ± *SE* or *edf* | *z* or *Χ^2^* | | *P* |
| --- | --- | --- | --- | --- | --- |
| *Spring* (qGAM1) | | | | | |
| 0.1 | Intercept | 79.8 ± 7.55 | 10.6 | <0.001 | |
|  | Temperature | -6.65 ± 2.06 | -3.23 | 0.001 | |
|  | ID | 0.38 | 0.38 | 0.32 | |
| 0.25 | Intercept | 90.1 ± 5.28 | 17.1 | <0.001 | |
|  | Temperature | -4.88 ± 1.85 | -2.64 | 0.008 | |
|  | ID | <0.001 | <0.001 | 0.54 | |
| 0.5 | Intercept | 102 ± 8.10 | 12.6 | <0.001 | |
|  | Temperature | -2.19 ± 2.85 | -0.77 | 0.44 | |
|  | ID | <0.001 | <0.001 | 0.97 | |
| 0.75 | Intercept | 117 ± 8.40 | 13.9 | <0.001 | |
|  | Temperature | 1.52 ± 2.81 | 0.54 | 0.59 | |
|  | ID | 0.05 | 0.12 | 0.13 | |
| 0.9 | Intercept | 130 ± 12.3 | 10.6 | <0.001 | |
|  | Temperature | 0.70 ± 3.23 | 0.22 | 0.83 | |
|  | ID | 0.46 | 2.61 | 0.02 | |
| *Autumn* (qGAM2) | | | | | |
| 0.1 | Intercept | 217 ± 28.8 | 7.55 | <0.001 | |
|  | Temperature | 1.72 ± 8.01 | 0.21 | 0.83 | |
|  | ID | 0.56 | 0.65 | 0.28 | |
| 0.25 | Intercept | 237 ± 19.8 | 12.0 | <0.001 | |
|  | Temperature | 5.16 ± 5.91 | 0.87 | 0.38 | |
|  | ID | <0.001 | <0.001 | 0.68 | |
| 0.5 | Intercept | 269 ± 17.5 | 15.3 | <0.001 | |
|  | Temperature | 2.45 ± 5.21 | 0.47 | 0.64 | |
|  | ID | <0.001 | <0.001 | 0.83 | |
| 0.75 | Intercept | 283 ± 19.2 | 14.8 | <0.001 | |
|  | Temperature | 2.54 ± 5.33 | 0.48 | 0.63 | |
|  | ID | 0.57 | 0.97 | 0.19 | |
| 0.9 | Intercept | 303 ± 16.8 | 18.0 | <0.001 | |
|  | Temperature | 6.60 ± 5.01 | 1.32 | 0.19 | |
|  | ID | 0.001 | <0.001 | 0.58 | |
