# Supplementary material for: The level of habitat patchiness influences movement strategy of moose in Eastern Poland
Source: PLoS One. 2020 Mar 19;15(3):e0230521. doi: 10.1371/journal.pone.0230521 (PMC7082038; doi:10.1371/journal.pone.0230521)

S1 Fig. Conceptual visualization of the five non-linear models fitted to net squared displacement (NSD) data. Each line represents different movement type.


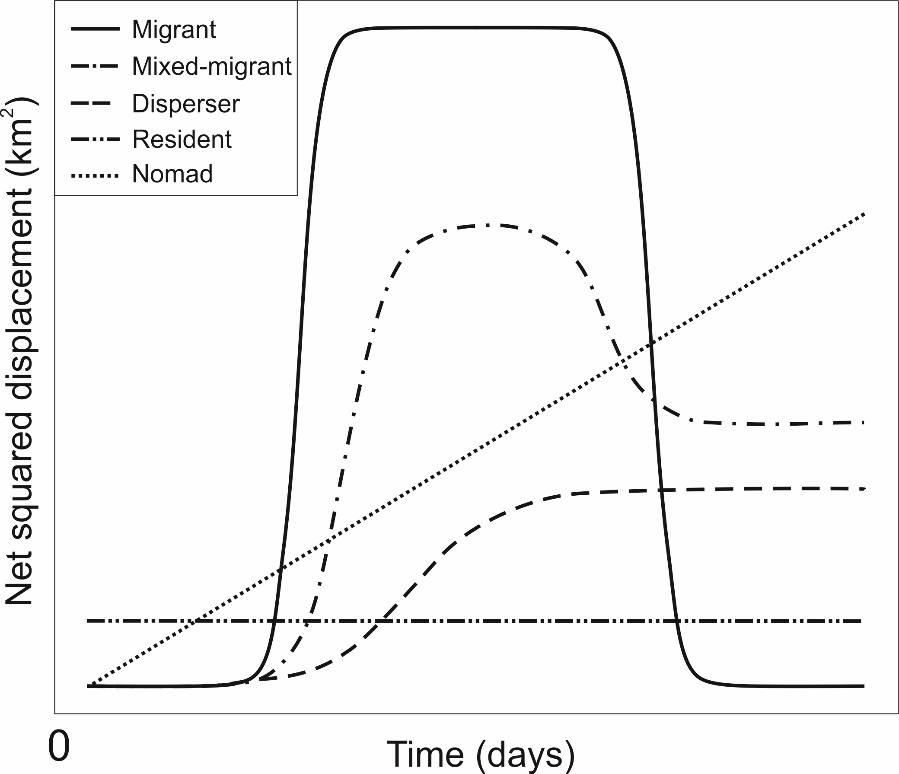

Supplement: S1 Fig — (DOCX) [file pone.0230521.s005.docx]
