# Supplementary material for: The level of habitat patchiness influences movement strategy of moose in Eastern Poland
Source: PLoS One. 2020 Mar 19;15(3):e0230521. doi: 10.1371/journal.pone.0230521 (PMC7082038; doi:10.1371/journal.pone.0230521)

S2 Fig. The predicted association between the time of departures of moose to summer ranges and the time of their returns to winter ranges in Biebrza.


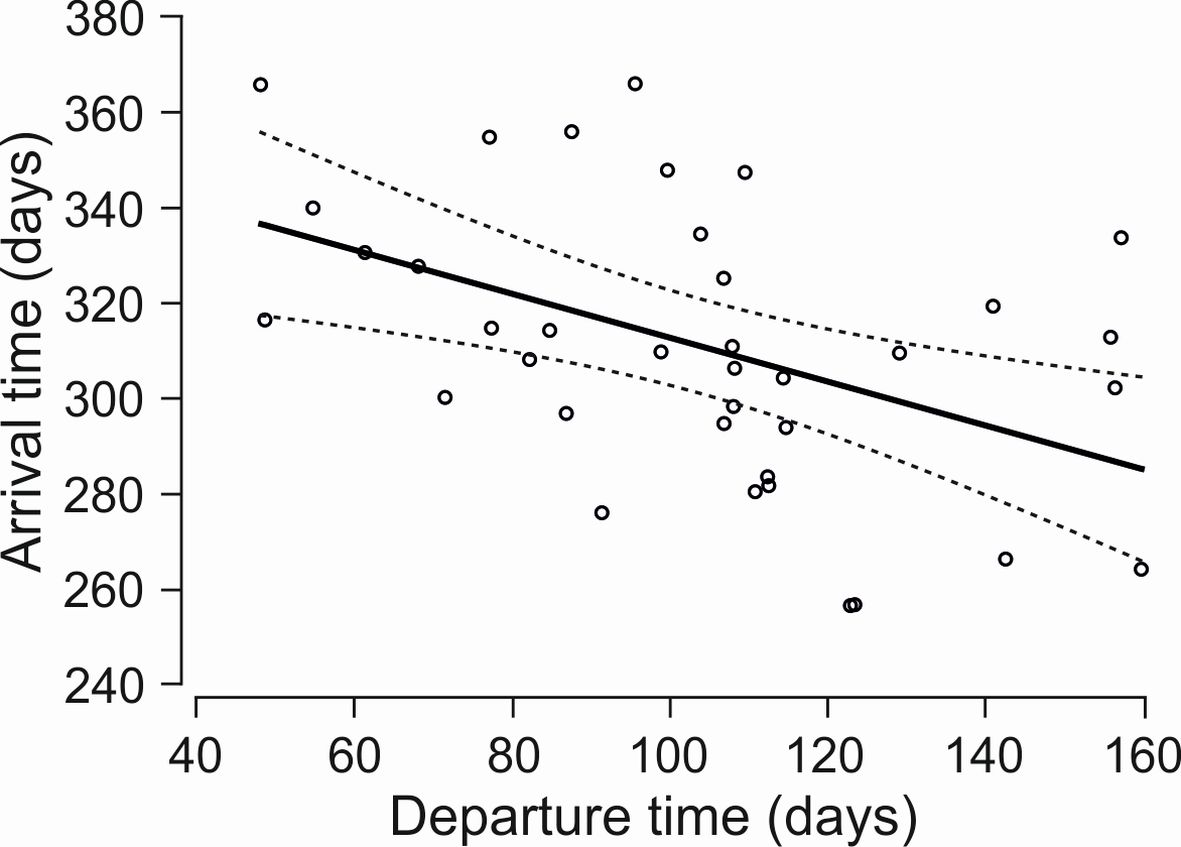

Supplement: S2 Fig — (DOCX) [file pone.0230521.s006.docx]
