# Supplementary material for: The level of habitat patchiness influences movement strategy of moose in Eastern Poland
Source: PLoS One. 2020 Mar 19;15(3):e0230521. doi: 10.1371/journal.pone.0230521 (PMC7082038; doi:10.1371/journal.pone.0230521)

Fig A2. The collation of weather data (snow cover and mean daily temperature) with the data on the start of migratory movement of moose in Biebrza. Migration time was derived from the non-linear models fitted to NSD data. Female 5F migrated twice between winter and summer home ranges.


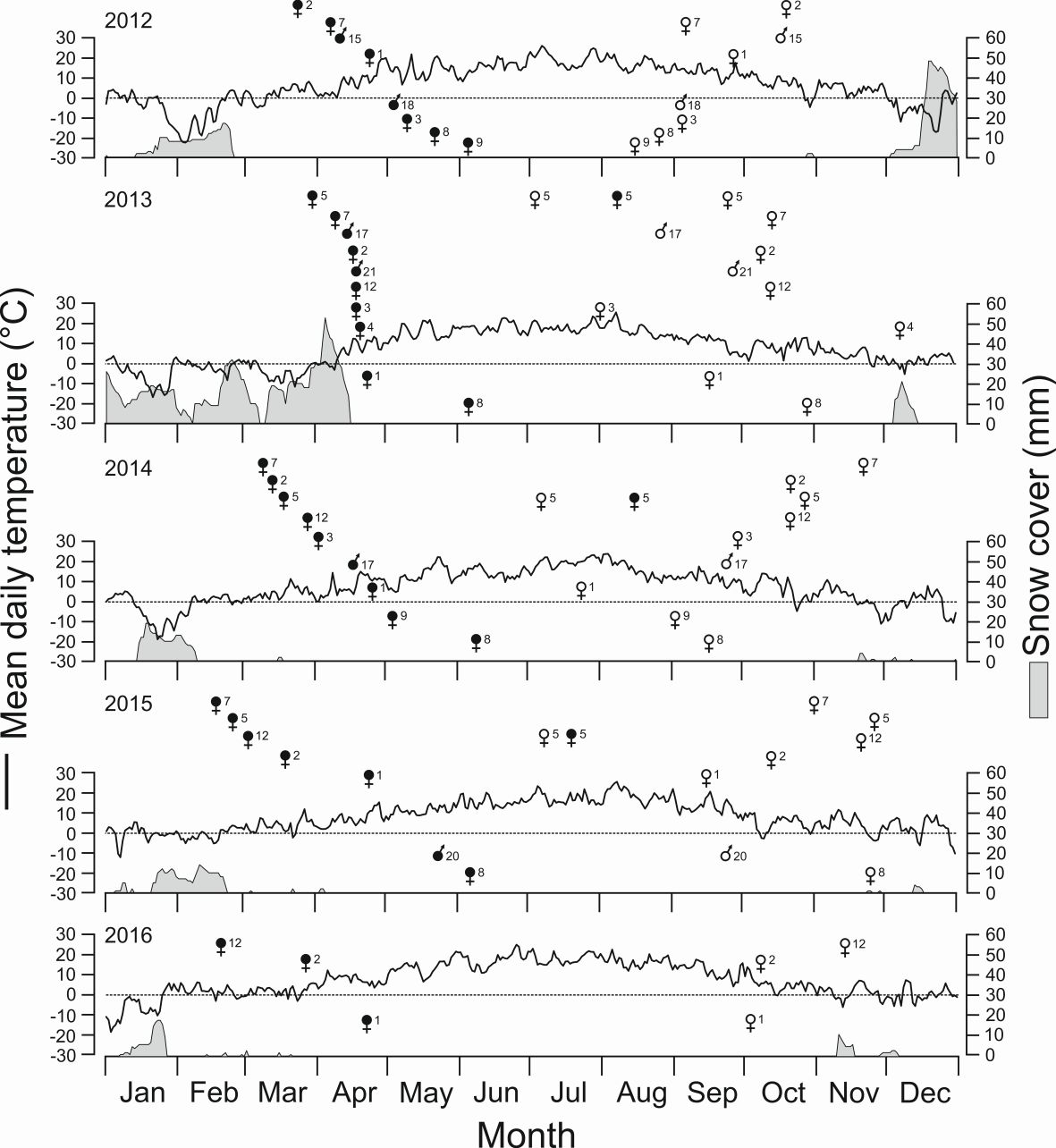

Supplement: S3 Fig — (DOCX) [file pone.0230521.s007.docx]
